# Development of a mouse iron overload-induced liver injury model and evaluation of the beneficial effects of placenta extract on iron metabolism

**DOI:** 10.1016/j.heliyon.2019.e01637

**Published:** 2019-05-10

**Authors:** Akihiro Yamauchi, Akiko Kamiyoshi, Takayuki Sakurai, Hiroyuki Miyazaki, Eiichi Hirano, Hong-Seok Lim, Taiichi Kaku, Takayuki Shindo

**Affiliations:** a Department of Cardiovascular Research, Shinshu University Graduate School of Medicine, Matsumoto, Japan; b Japan Bio Products Co., Ltd., Tokyo, Japan

**Keywords:** Molecular biology

## Abstract

Hepatic iron deposition is seen in cases of chronic hepatitis and cirrhosis, and is a hallmark of a poorer prognosis. Iron deposition is also found in non-alcoholic steatohepatitis (NASH) patients. We have now developed a mouse model of NASH with hepatic iron deposition by combining a methione- and choline-deficient (MCD) diet with an iron-overload diet. Using this model, we evaluated the effects of human placenta extract (HPE), which has been shown to ameliorate the pathology of NASH. Four-week-old male C57BL/6 mice were fed the MCD diet with 2% iron for 12 weeks. In liver sections, iron deposition was first detected around the portal vein after 1 week. From there it spread throughout the parenchyma. Biliary iron concentrations were continuously elevated throughout the entire 12-week diet. As a compensatory response, the diet caused elevation of serum hepcidin, which accelerates excretion of iron from the body. Accumulation of F4/80-positive macrophages was detected within the sinusoids from the first week onward, and real-time PCR analysis revealed elevated hepatic expression of genes related inflammation and oxidative stress. In the model mice, HPE treatment led to a marked reduction of hepatic iron deposition with a corresponding increase in biliary iron excretion. Macrophage accumulation was much reduced by HPE treatment, as was the serum oxidation-reduction potential, an index of oxidative stress. These data indicate that by suppressing inflammation, oxidative stress and iron deposition, and enhancing iron excretion, HPE effectively ameliorates iron overload-induced liver injury. HPE administration may thus be an effective strategy for treating NASH.

## 1 Introduction

Iron is an essential element in virtually all organisms, playing key roles in a variety of integrative metabolic pathways, including DNA-synthesis, hematopoiesis, mitochondrial biogenesis, energy metabolism and oxygen transport [1]. Iron deficiency causes anemia, while excess iron causes hemochromatosis. In the latter case, iron atoms cause Fenton reactions and promote production of toxic reactive oxygen species (ROS) [1, 2]. The liver, in particular, is susceptible to damage caused by ROS, and iron deposition in the liver is an exacerbating factor in cases of chronic hepatitis and cirrhosis [3].

Non-alcoholic fatty liver disease (NAFLD) is one of the most common liver conditions seen in outpatient practice [4] and is strongly associated with metabolic syndrome and insulin resistance [5]. The spectrum of NAFLD includes the relatively benign “simple steatosis” and the more severe “non-alcoholic steatohepatitis” (NASH). NASH is broadly defined as the presence of steatosis with inflammation and progressive fibrosis [6, 7]. It has been shown that NASH ultimately leads to cirrhosis and hepatocellular carcinoma in 15–25% of the patients [8, 9, 10]. Hepatic iron deposition has been confirmed in about one-third of adult NAFLD patients and is a hallmark of a poorer prognosis [11].

For more than 40 years, human placental extract (HPE) has been prescribed clinically to treat chronic hepatitis, cirrhosis and other hepatic diseases. In experimental animal models of hepatitis, HPE reportedly ameliorates hepatic injury mediating liver regeneration and inhibiting inflammatory reactions and hepatocyte apoptosis [12, 13]. Moreover, Shimokobe et al. reported that HPE is effective in NASH patients who are unresponsive to lifestyle intervention [14]. Those patients were treated for 8 weeks with Laennec, a HPE formulation, which produced significant reductions in serum transaminases (AST and ALT).

In an earlier study (Heliyon 2017), we developed a mouse NASH model by feeding the mice a methione- and choline-deficient (MCD) diet with high-salt loading (8% NaCl in the drinking water) for 5 weeks in heterozygous RAMP2 knockout mice (RAMP2+/−) [15]. Using this model, we evaluated the effects of HPE-treatment. Serum levels of AST and ALT were reduced in the HPE-treated group, as was hepatic expression of TNF-α and MMP9, which is indicative of reductions in the severity of hepatic inflammation and tissue remodeling. HPE treatment also diminished oxidative stress. Because it is safe and well tolerated, use of HPE is a potentially effective approach to the treatment of NASH.

In the present study, we developed a mouse model of NASH with hepatic iron deposition by combining the MCD diet with iron-overload. Focusing particularly on iron metabolism, we evaluated the effects of HPE-treatment.

## 2 Materials and methods

### 2.1 Animals

Four-week-old wild-type C57BL/6J male mice were purchased from a supplier of experimental animals (Charles river laboratories Japan, Inc. Kanagawa, Japan) and used for the study. All mice were maintained according to a strict procedure under specific pathogen-free conditions in an environmentally controlled (12-h light/dark cycle; room temperature, 22 ± 2C) breeding room at the Division of Laboratory Animal Research, Department of Life Science, Research Center for Human and Environmental Sciences, Shinshu University. Before the operative procedures, the mice were anesthetized through intraperitoneal injection of a combination anesthetic that included 0.3 mg/kg of medetomidine (Nippon Zenyaku Kogyo Co.Ltd., Koriyama Japan), 4.0 mg/kg of midazolam (Astellas Pharma Inc. Tokyo Japan) and 5.0 mg/kg of butorphanol (Meiji Seika Pharma Co.Ltd., Tokyo Japan). All animal experiments were conducted in accordance with the ethical guidelines of Shinshu University.

### 2.2 Diet and HPE treatment

The MCD with 2% carbonyl iron (MCD-Fe) diet was custom-made for this study (Oriental Yeast Co., Ltd, Tokyo, Japan). The components of the diet are shown in Table 1. The normal diet (AIN-93G, Oriental Yeast) included methionine and choline without carbonyl iron. The diet was administered for 12 weeks, beginning when the mice were 4 weeks old.

**Table 1:** Components of the diets.

Elements of diet	Normal diet (AIN-93G)	MCD with 2% iron diet
Amino acid mix (without methionine)	-	17.83%
Amino acid mix (with methionine)	18.34%	-
Sucrose	10.00%	10.00%
Lard	10.00%	10.00%
Cellulose	5.00%	5.00%
AIN-93 vitamin mix (without choline)	-	1.00%
AIN-93 vitamin mix (with choline)	1.00%	-
AIN-76 mineral mix	3.50%	3.50%
Tertiary butylhydroquinone	0.002%	0.002%
Corn starch	38.988%	25.948%
Pregelatinized corn starch	13.17%	15.17%
Fe-citrate (Fe17%)	-	11.55%

The HPE used in this study was hydrolysate of human placenta (Laennec; Japan Bio Products Co., LTD, Tokyo, Japan). Mice were intramuscularly administered 0.1 ml of Laennec (3.6 mg/kg) or control saline twice a week during the MCD-Fe diet. Body weights were measured every day at around 10:00 am.

### 2.3 Histology

Tissues were fixed overnight in 10% formalin, embedded in paraffin, and cut into 5-μm-thick sections for histological examination. The specimens were then deparaffinized for Berlin blue staining. For immunohistochemical analysis, rat anti-mouse F4/80 antibody (BIO-RAD, Hercules, CA) and rat anti-mouse 4-hydroxy-2-nonenal (4HNE) (NOF Corporation, Tokyo, Japan) were used. DAPI (Thermo Fisher Scientific, MA) was used to stain the nuclei. Sections were observed using a KEYENCE model BZ-X710 microscope (Osaka, Japan). The area of interest was quantified using the BZ-H3C module with the BZ-X710 microscope (KEYENCE).

### 2.4 Quantitative real-time RT-PCR analysis

Total RNA was isolated from liver samples using a PureLink RNA Mini Kit (Thermo Fisher Scientific). RNA quality was then verified using electrophoresis, and concentrations were measured using an Oubit 3.0 fluorometer (Thermo Fisher Scientific). Thereafter, the extracted RNA was treated with DNA-Free (Thermo Fisher Scientific) to remove contaminating DNA, and 2-μg samples were subjected to reverse transcription using a PrimeScript™ RT reagent Kit (Takara Bio, Shiga, Japan). Quantitative real-time RT-PCR was carried out using a StepOne Plus Real-Time PCR System (Thermo Fisher Scientific) with SYBR green (Toyobo, Osaka, Japan) or Realtime PCR Master Mix (Toyobo). Values were normalized to mouse GAPDH expression (Pre-Developed TaqMan assay reagents, Thermo Fisher Scientific). The primers used are listed in Table 2.

**Table 2:** Primers used for real-time PCR.

IL-1β	Forward	CTACAGGCTCCGAGATGAACAAC
	Reverse	TCCATTGAGGTGGAGAGCTTTC
IL-6	Forward	CTGCAAGAGACTTCCATCCAGTT
	Reverse	GAAGTAGGGAAGGCCGTGG
p67 phox	Forward	CAGACCCAAAACCCCAGAAA
	Reverse	AAAGCCAAACAATACGCGGT
p47phox	Forward	ATCCTATCTGGAGCCCCTTGA
	Reverse	CACCTGCGTAGTTGGGATCC
p22 phox	Forward	GGCCATTGCCAGTGTGATCT
	Reverse	GCTCAATGGGAGTCCACTGC
collagen α1	Forward	ATGGATTCCCGTTCGAGTACG
	Reverse	TCAGCTGGATAGCGACATCG

### 2.5 Measurement of iron concentrations in bile and urine

After laparotomy, the gallbladder was removed, and the bile within it was collected and frozen at −80 °C. Spot urine was collected and frozen in the same way. Iron concentrations in the bile and urine samples were determined using a Metal assay ELISA kit (Metallogenics Co., Ltd., Chiba, Japan).

### 2.6 Measurement of hepcidin concentrations in serum

Blood samples were collected from the abdominal aorta using a 22G needle. The collected samples were stored on ice for about 30 min and allowed to clot, after which they were centrifuged twice at 3,500 rpm for 10 min at 4 °C, and the serum was collected. Serum hepcidin concentrations were measured at Medical Care Proteomics Biotechnology Co., Ltd. (Ishikawa, Japan).

### 2.7 Measurement of serum oxidation stress

Serum oxidation-reduction potentials were measured using a RedoxSYS Analyzer (Aytu Bioscience, CO).

### 2.8 Statistical analysis

Values are expressed as means ±SEM. Student's t test was used to evaluate differences. Values of p < 0.05 were considered significant.

## 3 Results

### 3.1 Changes of body weight on the MCD with iron-overload diet

**Fig. 1:**
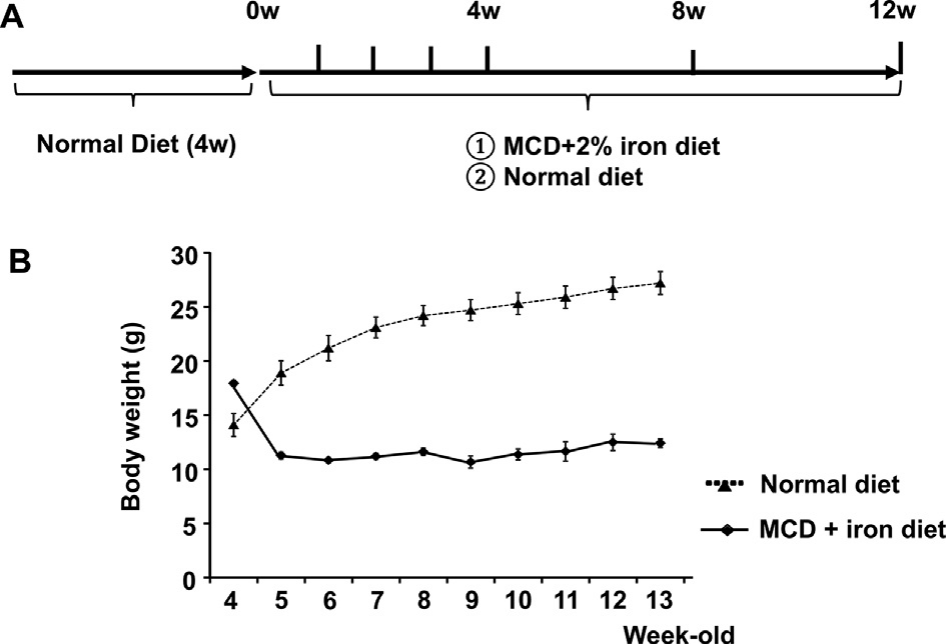
Protocol used for the methione- and choline-deficient (MCD) with iron-overload diet. A, Schematic depiction of the protocol. After the 4 weeks of a normal diet, the mice were divided into MCD +2% iron (MCD-Fe) diet and normal diet groups. The two diets were then administered for 12 weeks. B, Body weight changes on the MCD-Fe and normal diets. Body weights were measured in 4- to 13-week-old mice. Symbols depict means ±SEM; n = 6.

The MCD-Fe or normal diet was administered for 12 weeks, beginning when the mice were 4 weeks old (Fig. 1A). We found that mice in the MCD-Fe group lost a substantial amount of weight during the first week of the diet (Fig. 1B). After 1 week, the weights of mice on the MCD-Fe diet were about 70% of the weights of mice on the normal diet. Thereafter, the body weights of mice on the MCD-Fe diet stabilized and remained relatively constant, while the weights of mice on the normal diet gradually increased.

### 3.2 Iron deposition in the liver

**Fig. 2:**
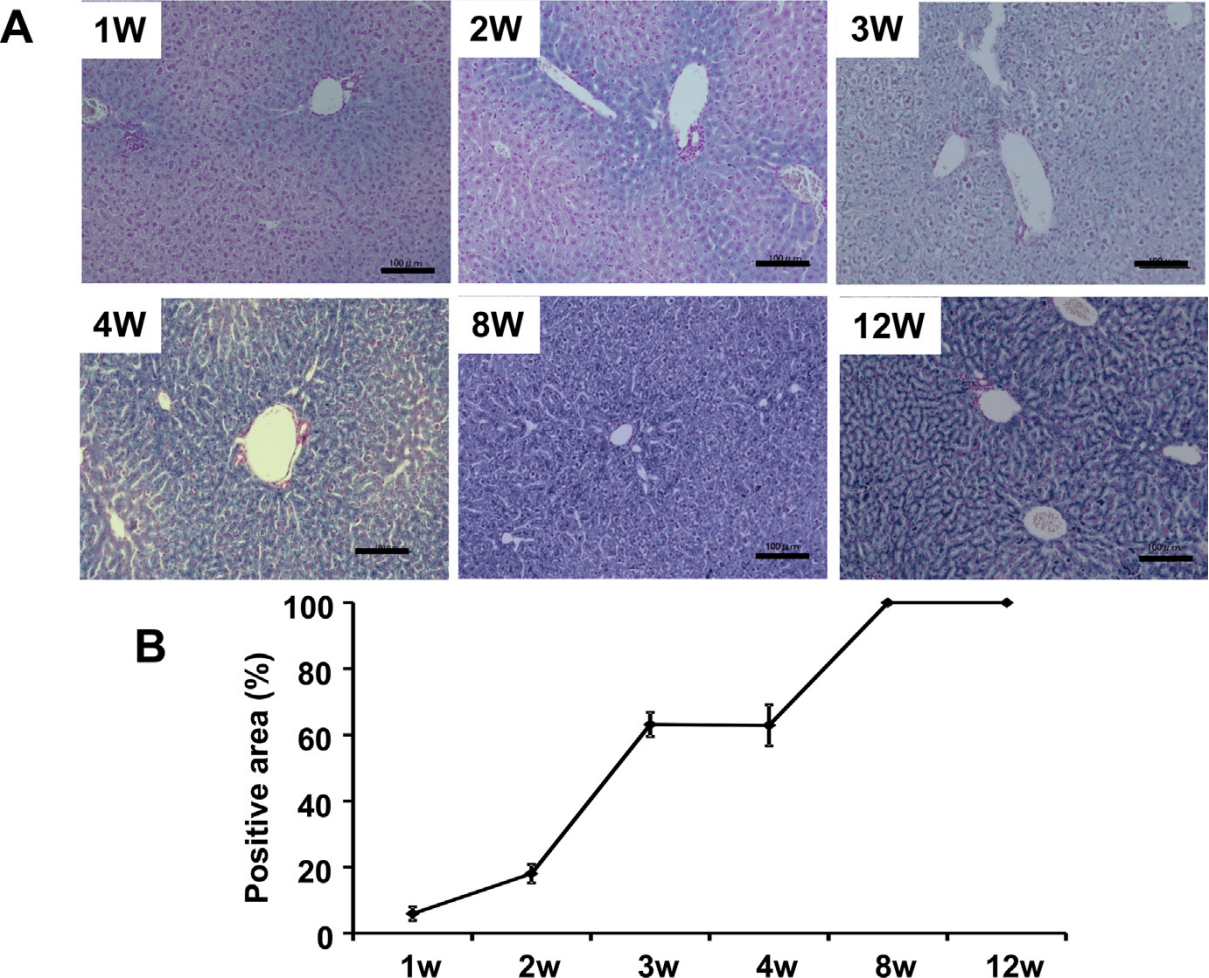
Evaluation of iron deposition in the liver. A, Liver sections from mice fed the MCD-Fe diet. Shown is Berlin blue staining of the sections. Iron deposits appear blue. Note the progressive increase in blue staining. Scale bars = 100 μm. B, Percent Berlin blue-positive area within the liver sections. Symbols depict means ±SEM; n = 6.

No iron deposition was detected in mice on the normal diet (data not shown). Liver samples collected from mice after 1–4, 8 and 12 weeks on the MCD-Fe diet were sectioned and examined (Fig. 2A). Areas positive for Berlin blue staining, which is indicative of iron deposition, were calculated (Fig. 2B). Iron deposition was already detectable at the periphery of lobules (Zone 1, portal triads) after 1 week, and subsequently spread into the liver parenchyma. At 4 weeks, the accumulation of iron became prominent, and sporadic dense deposits were noticed around the portal vein. By 8 weeks, ballooning degeneration of hepatocytes was prominent, and the iron deposition had spread toward the central vein. At 12 weeks, the iron deposition had increased further and was detected throughout the hepatic lobules.

### 3.3 Iron excretion and serum hepcidin changes in mice on the MCD with iron-overload diet

**Fig. 3:**
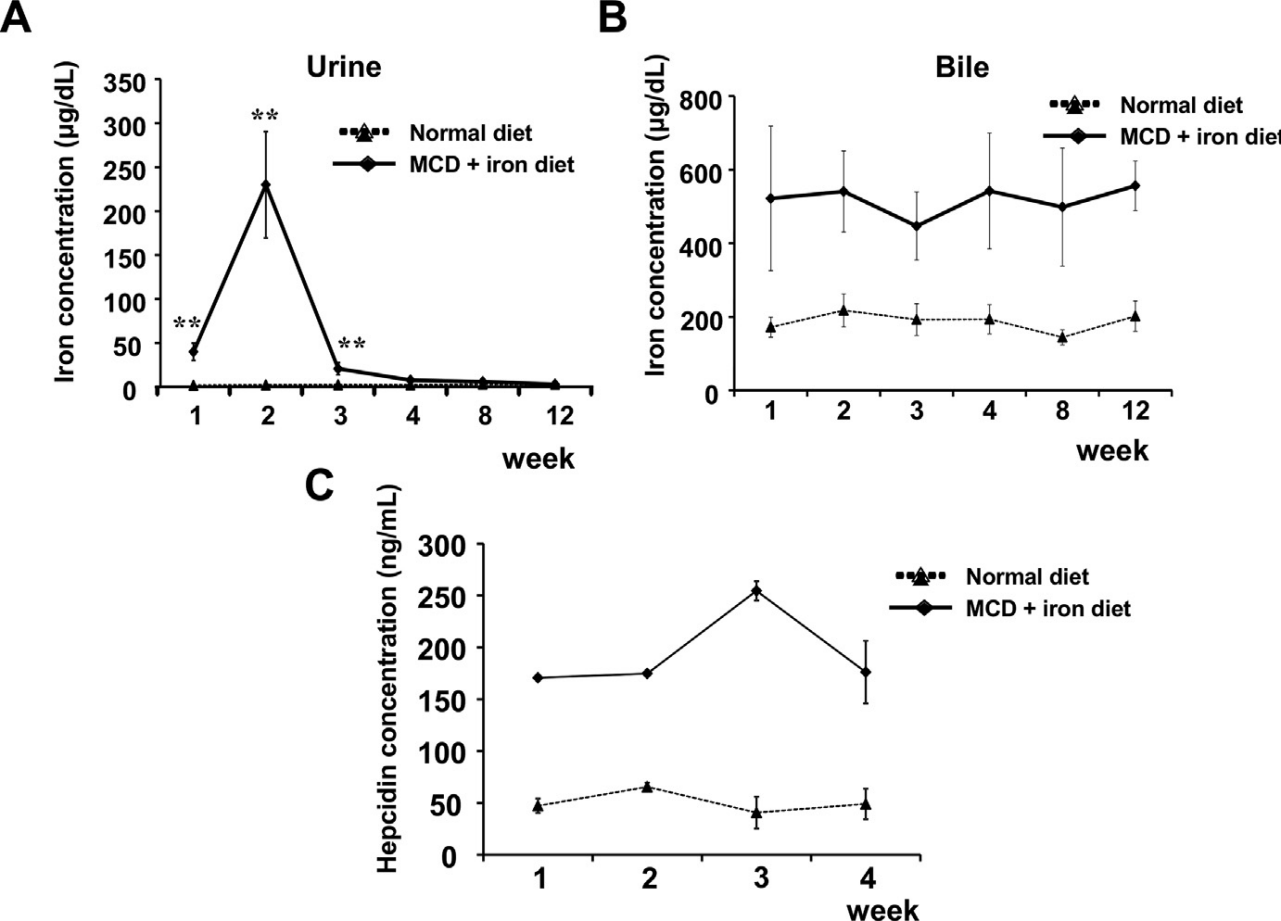
Evaluation of iron excretion and serum hepcidin level. A, B, Time-dependent changes in urinary (A) and biliary (B) iron concentrations in mice fed the MCD-Fe or normal diet. Symbols depict means ±SEM; n = 5–9. **p < 0.01, MCD-Fe vs. control. C, Serum hepcidin concentrations measured during the first 4 weeks of the MCD-Fe and normal diets. Symbols depict means ±SEM; n = 9 in the MCD-Fe group, and n = 2 in the normal group.

We evaluated iron excretion via the kidney and liver. In the normal diet group, the average urinary iron concentration was 2.37 ± 1.09 μg/dL during the study. In the MCD-Fe group, the iron concentration in urine was significantly elevated to 39.94 ± 9.83 μg/dL after 1 week and reached 229.94 ± 60.50 μg/dL after 2 weeks (Fig. 3A). Thereafter, urinary iron concentrations in MCD-Fe mice declined to the control level.

Iron is also discharged from the liver into the intestine with the bile and discarded in the feces. In mice on the normal diet, the average biliary iron concentration was 187.01 ± 36.16 μg/dL. In mice in the MCD-Fe group, the iron concentration in bile was continuously elevated throughout the study (446.92 ± 92.68 μg/dL to 556.25 ± 202.00 μg/dL) (Fig. 3B).

The peptide hormone hepcidin is the key regulator of iron metabolism in mammals, acting to accelerate iron excretion from the body. We found that in mice on the MCD-Fe diet, serum hepcidin levels were continuously elevated throughout the study (Fig. 3C). On the normal diet, the average serum hepcidin level during the study was 52.47 ± 9.16 ng/ml. In the MCD-Fe mice, serum hepcidin levels ranged from 170.68 ± 0.49 ng/ml (1 week) to 254.5 ± 9.26 ng/ml (3 weeks). The elevation of serum hepcidin is thought be a compensatory response to the iron-overload. The temporal pattern of the hepcidin elevation appeared to parallel that of the biliary iron concentration. This suggests hepcidin was upregulated in response to the hepatic iron accumulation.

### 3.4 Macrophage accumulation in the liver

**Fig. 4:**
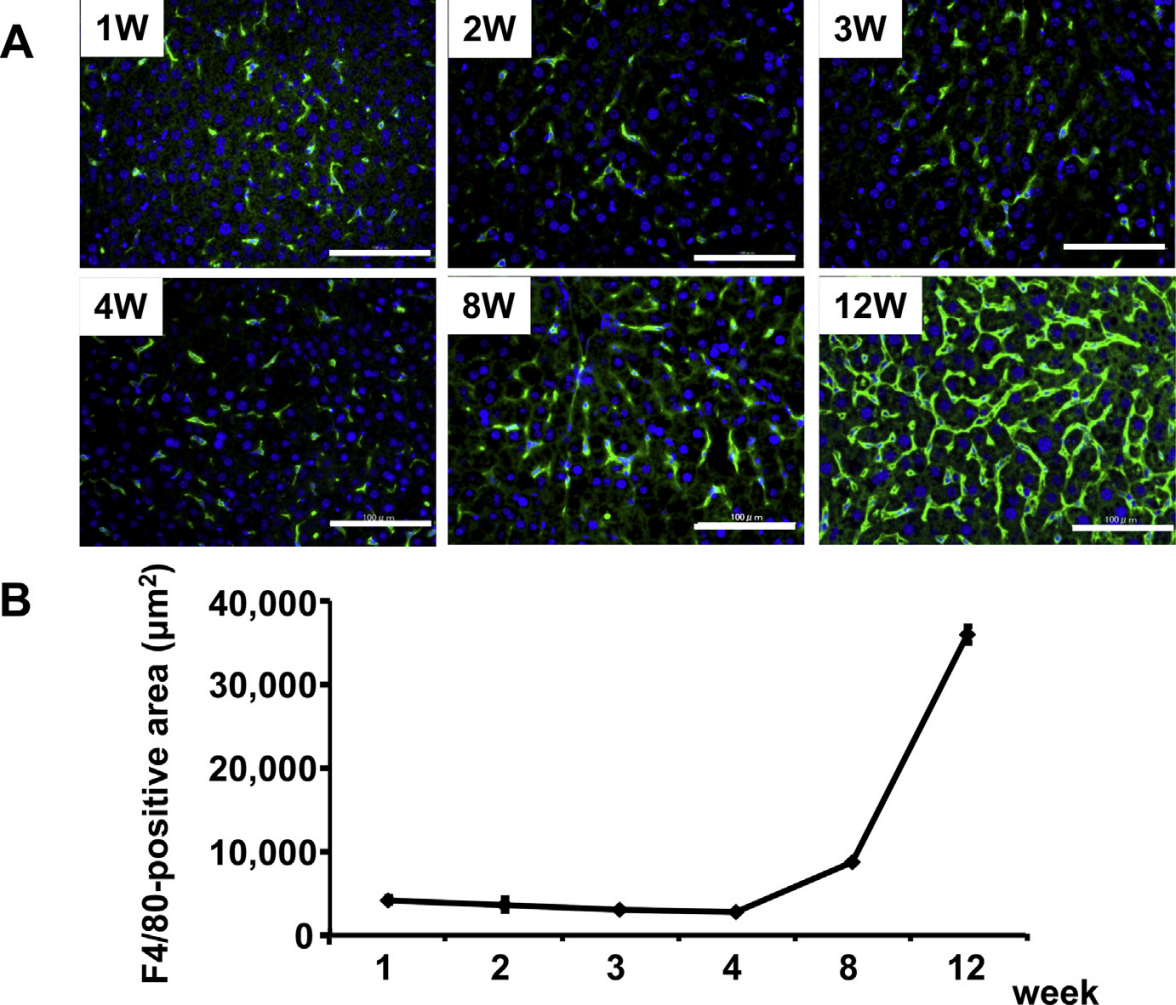
Evaluation of macrophage accumulation in the liver. A, Immunostaining of F4/80 in sections of liver samples collected from mice on the MCD-Fe diet for the indicated times. Green; F4/80, Blue; DAPI. Scale bars = 100 μm. Note the time-dependent increase in green fluorescent staining. B, Calculation of F4/80-positive area per 200× microscope field. Symbols depict means ±SEM; n = 6.

To assess macrophage accumulation, we analyzed F4/80 immunostaining in liver sections. After 1 week on the MCD with iron-overload diet, F4/80-positive macrophages were detected along the sinusoids (Fig. 4A). At that time, the F4/80-positive area per 200× microscope field was 4,211 ± 318.5 μm^2^ (Fig. 4B). Macrophage accumulation then spread from the portal triad to peripheral regions, following a distribution pattern similar to that of hepatic iron deposition. The extent of macrophage accumulation did not change from week 1–4; however, it showed marked elevation at 8 and 12 weeks. By 8 weeks, the F4/80-positive area was 8,730 ± 970.5 μm^2^, and it increased to 35,926 ± 44.0 μm^2^ by 12 week.

### 3.5 Inflammation, oxidative stress and fibrosis-related gene expression in the liver

**Fig. 5:**
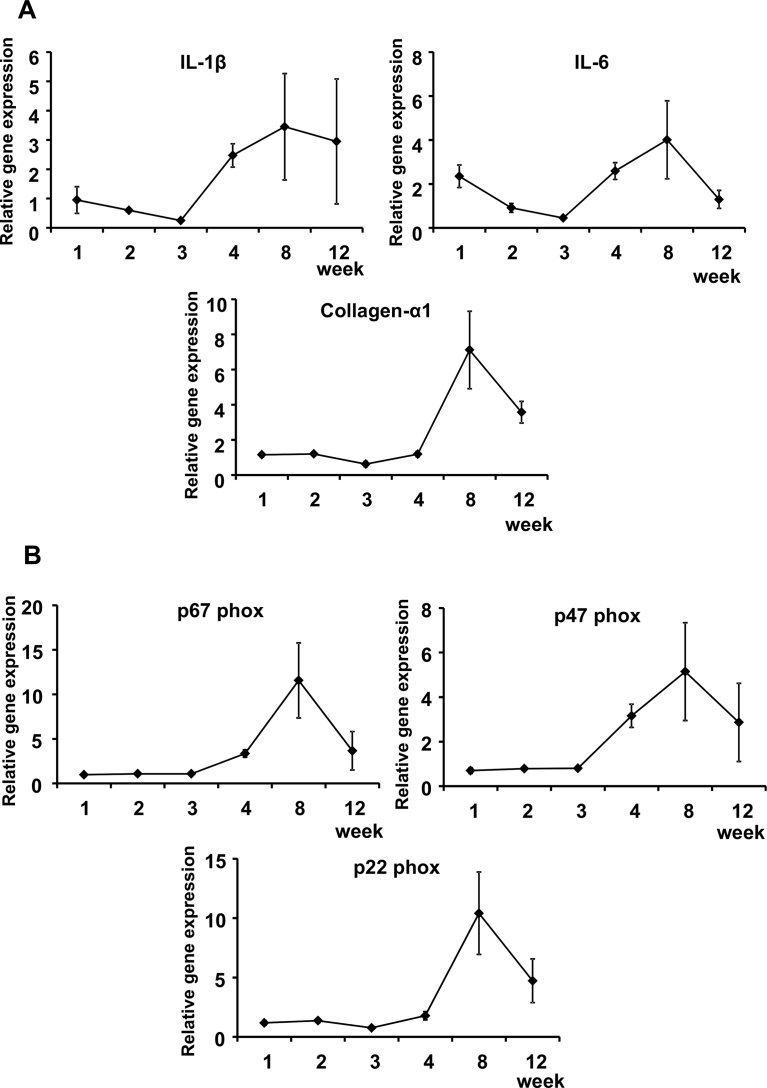
Inflammation-, fibrosis- and oxidative stress-related gene expression in liver. A, Time courses of the relative hepatic expression levels of genes encoding the proinflammatory cytokines IL-1β and IL-6 and the fibrosis-related molecule collagen-α1 in mice on the MCD-Fe diet. B, Time courses of the relative hepatic gene expression of NADPH oxidase subunits. Symbols depict means ±SEM; n = 4.

Real-time PCR analysis of the liver revealed elevated expression of genes related to inflammation, oxidative stress and fibrosis in mice in the MCD-Fe group. Expression of the proinflammatory cytokines IL-1β and IL-6 reached a peak at 8 weeks, as did expression of collagen-α1 (Fig. 5A). Expression of the NADPH oxidase subunits p67 phox, p47 phox and p22 phox was also elevated in MCD-Fe mice, and they continued to be elevated at week 12 (Fig. 5B).

### 3.6 HPE-treatment increases iron excretion and suppresses hepatic iron deposition

**Fig. 6:**
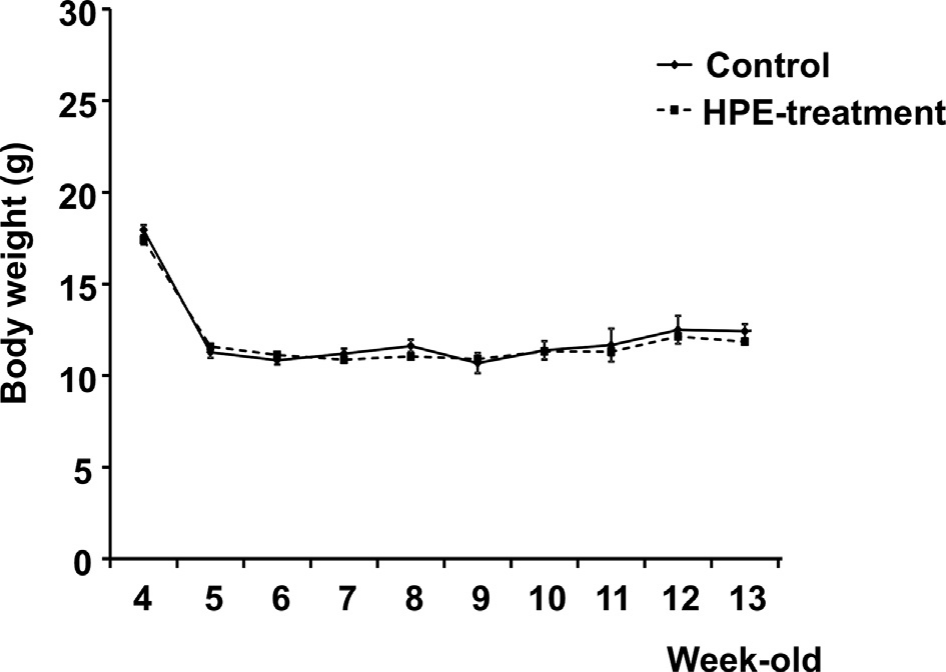
Effect of HPE treatment on body weights in mice on the MCD-Fe diet. Body weights were measured in 4- to 13-week-old mice fed the MCD-Fe diet with or without HPE treatment. Symbols depict means ±SEM. n = 6.

**Fig. 7:**
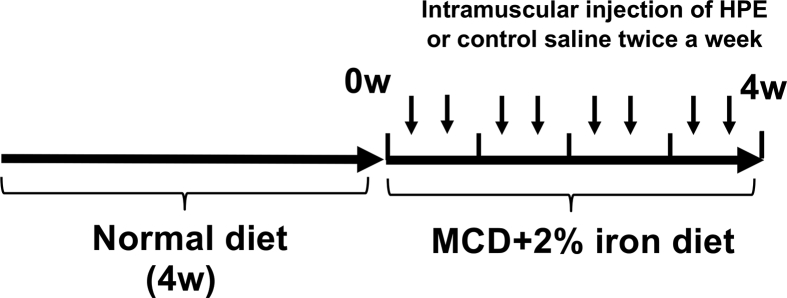
Schematic depiction of the protocol used for HPE administration to mice on the MCD-Fe diet. After 4 weeks of normal diet, the mice were administered the MCD +2% iron diet for 4 weeks. Mice were intramuscularly administered HPE (0.1 ml of Laennec (3.6 mg/kg)) or control saline twice a week during the 4 weeks.

**Fig. 8:**
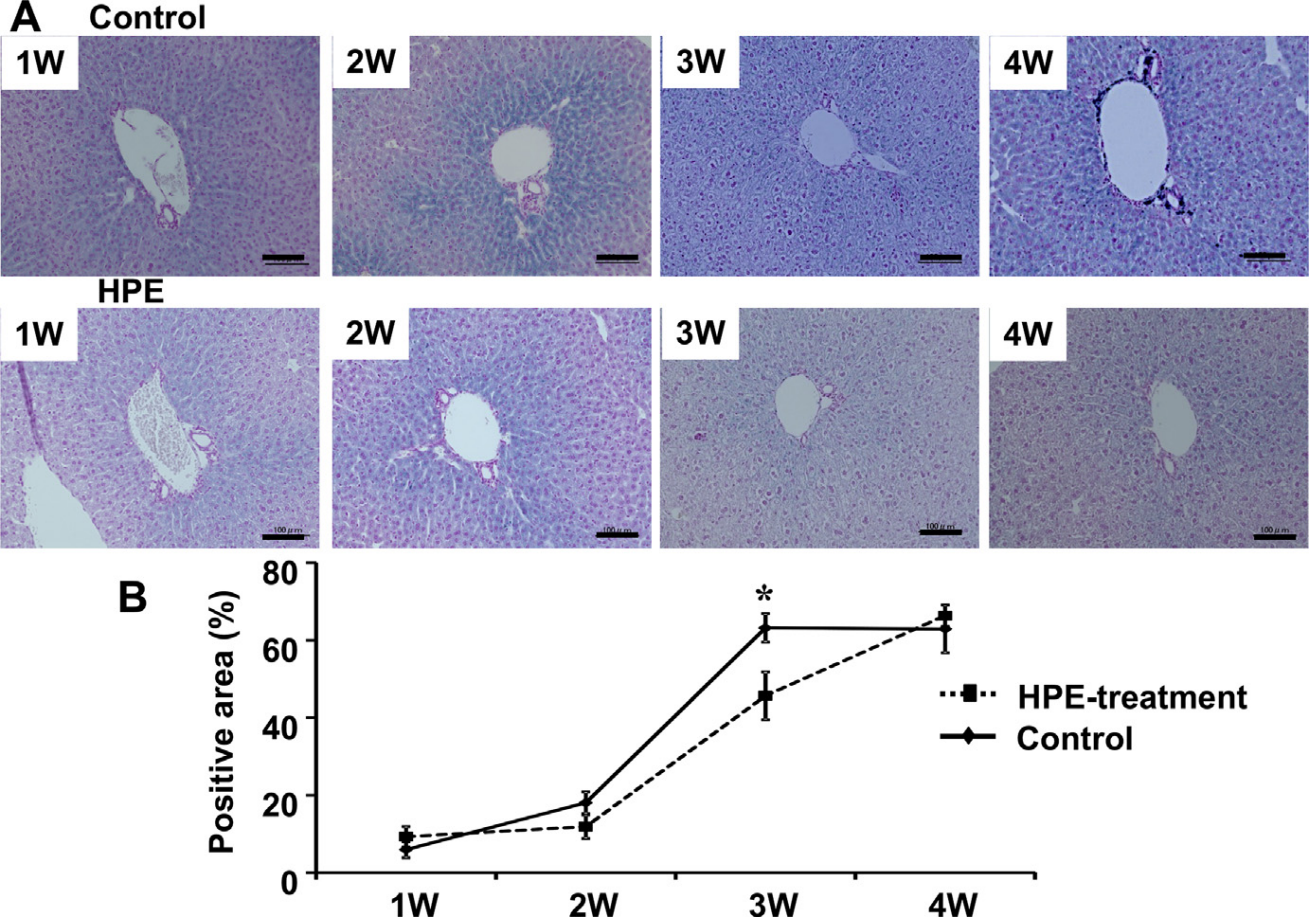
Effect of HPE treatment on iron deposition in the liver. A, Sections of liver samples collected from mice on the MCD-Fe diet for the indicated times, with or without HPE treatment. The sections are stained with Berlin blue. Scale bars = 100 μm. B, Percent Berlin blue-positive area within the liver sections. Symbols depict means ±SEM; n = 6. *p < 0.05.

**Fig. 9:**
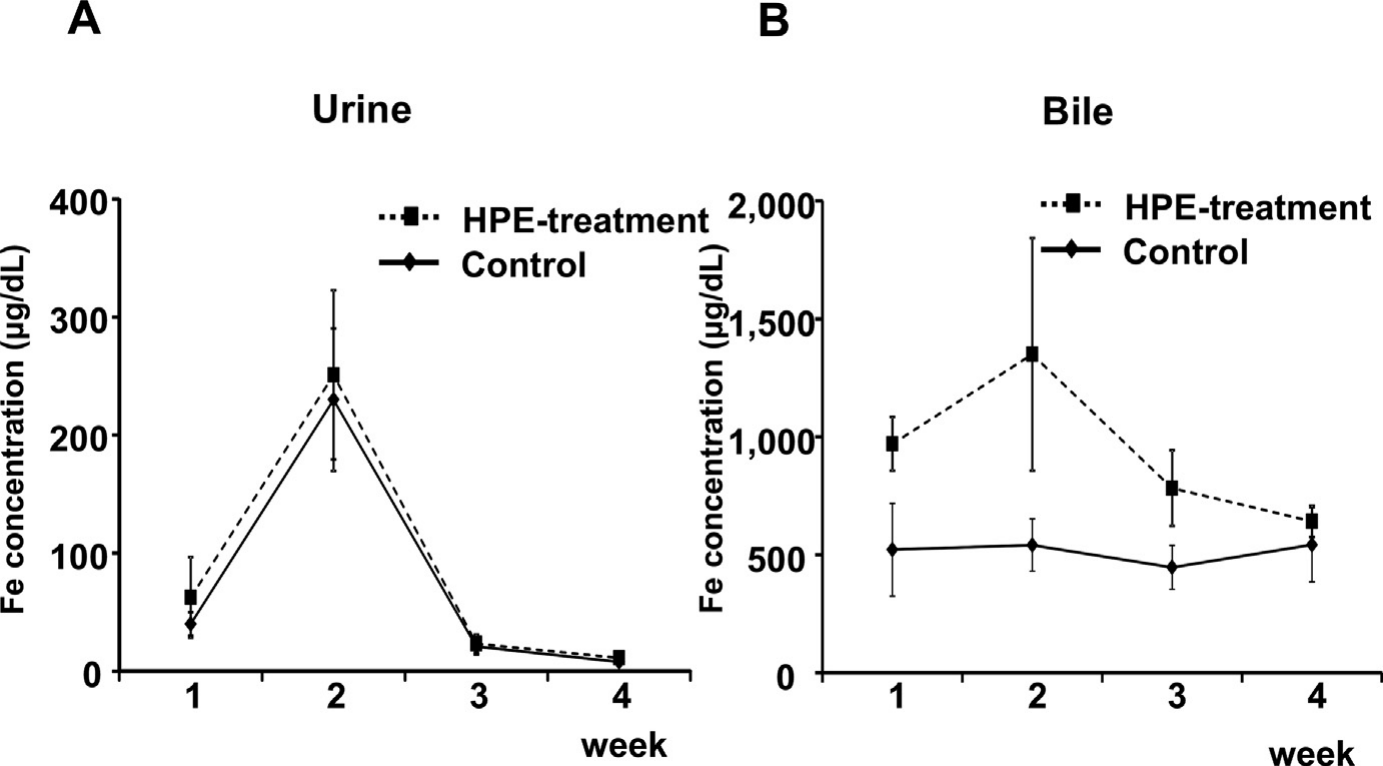
Effect of HPE treatment on urinary and biliary iron excretion. Time-dependent changes in urinary (A) and biliary (B) iron concentration in mice on the MCD-Fe diet with or without HPE treatment. Symbols depict means ±SEM. n = 5.

**Fig. 10:**
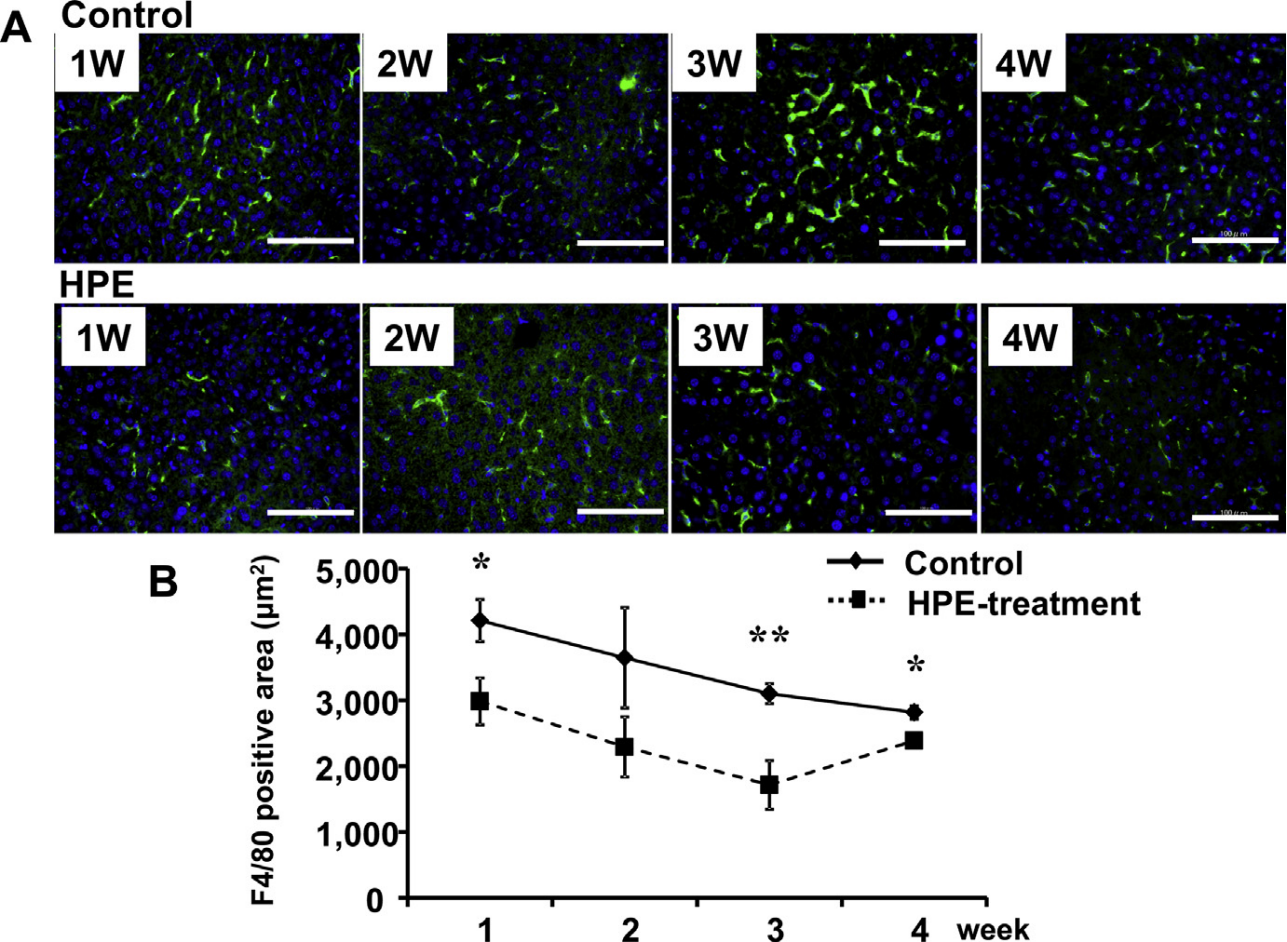
Effect of HPE-treatment on macrophage accumulation in the live. A, Immunostaining of F4/80 in sections of liver samples collected from mice on the MCD-Fe diet for the indicated times, with or without HPE treatment. Green, F4/80; Blue, DAPI. Scale bars = 100 μm. B, Calculation of F4/80-positive area per 200× microscope field. Symbols depict means ±SEM; n = 6. *p < 0.05, **p < 0.01, HPE treated vs. untreated.

HPE had no effect on the body weight changes seen in MCD-Fe mice (Fig. 6). Using our mouse model of diet-induced NASH with hepatic iron deposition, we evaluated the effects of HPE treatment (Fig. 7). Because the liver injury was severe and intractable after 8 or 12 weeks on the MCD-Fe diet, we evaluated the effect of HPE during weeks 1–4. HPE treatment resulted in a significant reduction in hepatic iron deposition at week 3 (Fig. 8A, B). The urinary iron concentration reached its peak at 2 weeks in both the HPE-treated and untreated groups, and there was no difference between the two groups (Untreated: 229.94 ± 60.50 μg/dl vs. HPE: 250.84 ± 71.76 μg/dl) (Fig. 9A). By contrast, peak biliary iron excretion was increased by HPE treatment (Untreated: 540.73 ± 110.38 μg/dl vs. HPE: 1,349.74 ± 493.93 μg/dl) (Fig. 9B). F4/80-positive macrophage accumulation was also much reduced by HPE treatment (Fig. 10A). In HPE-treated mice, the F4/80-positive area was reduced to 70% of that in untreated mice after week 1, and it fell to 50% of that in untreated mice by week 3 (Fig. 10B).

### 3.7 HPE suppresses oxidative stress in the NASH with iron deposition model

**Fig. 11:**
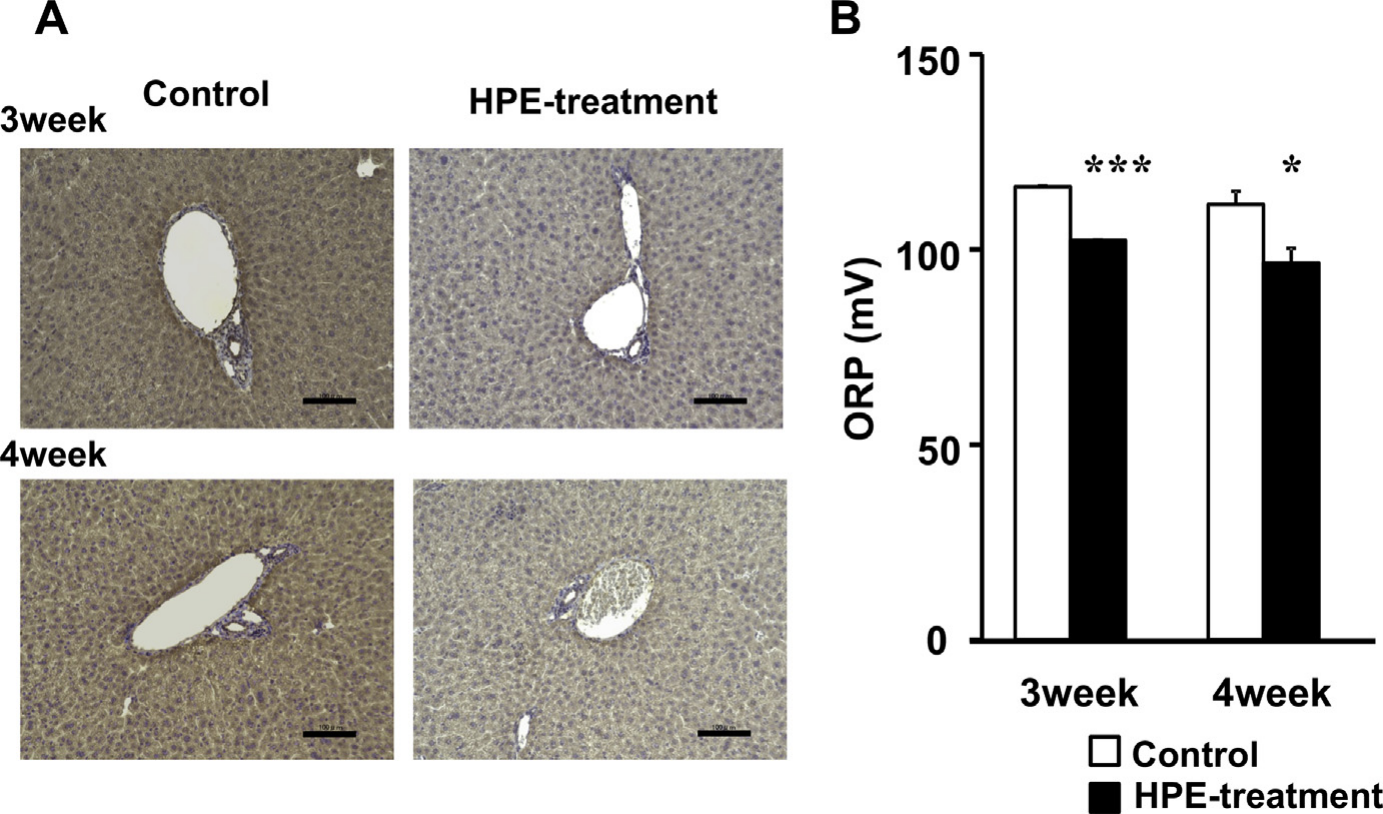
Effect of HPE treatment on oxidative stress. A, Immunostaining of 4HNE in sections of liver samples collected from mice on the MCD-Fe diet for the indicated times, with or without HPE treatment. Scale bars = 100 μm. B, Serum oxidation-reduction potentials (ORP) were measured as an index of oxidative stress using a RedoxSYS Analyzer. Bars depict means ±SEM; n = 4–5. *p < 0.05, ***p < 0.001, HPE treated vs. untreated.

Finally, we evaluated the oxidative stress level in the NASH with iron deposition model. Control groups showed strong immunostaining of 4-hydroxy- 2-nonenal (4HNE), a product of lipid peroxidation, at weeks 3 and 4 of the experiment. However, the staining was reduced by HPE-treatment (Fig. 11A). We then analyzed the serum oxidative stress level using the oxidation-reduction potential (ORP) as an index. We found that HPE treatment significantly reduced the level of oxidative stress from 116.1 ± 0.20 mV to 102.3 ± 0.10 mV after week 3 and from 111.5 ± 3.36 mV to 96.6 ± 3.73 mV after week 4 (Fig. 11B).

## 4 Discussion

There are currently no standard animal models that correctly reproduce the pathogenesis of NASH in humans, though several have been proposed [16]. Dietary NASH models are useful for mimicking the pathogenesis of diet-induced obesity and the resultant metabolic disturbances. However, whereas long-term intake of a high-fat diet leads to obesity and fatty liver in mice, it does not evoke liver fibrosis, which is a defining histological feature of NASH. In the MCD diet model, fibrosis started from the region adjacent to the sinusoids [15]. Liver sinusoidal endothelial cells (LSECs) are the front-line exposed to the various metabolites and chemicals that enter the liver in the circulation. They exhibit greater endocytotic activity than other types of endothelial cells [17], and they express scavenger receptors, such as the mannose receptor, Fc-receptor and stabilin-2, which may protect the parenchymal hepatocytes by scavenging toxic molecules. This may explain why hepatic injury reportedly begins with damage to LSECs [18, 19]. Because the MCD diet model in mice closely replicates the histological features of the fibrosis observed in human NASH, we selected that model for evaluation of the pathophysiology of NASH in our earlier study [15].

Iron deposition can be detected in cases of chronic organ dysfunction, including heart failure, renal failure and liver failure [20, 21]. Deposition of excess iron causes production of ROS, which can have toxic effects on nucleic acids, proteins and lipids [2]. Although, the liver plays a central role in iron homeostasis, excess iron deposition is an exacerbating factor in cases of liver failure [22]. ROS derived from the iron deposition secondarily generate peroxidized lipids and proteins and reduces hepatic anti-oxidant levels and nitric oxide production, all of which cumulatively damage hepatocytes [23]. Iron deposition-induced liver damage is most apparent in hereditary hemochromatosis, which is caused by a genetic abnormality of iron metabolism-related factors. In cases of hereditary hemochromatosis, excess iron deposition in the liver causes hepatocyte death and fibrosis, which ultimately results in liver cirrhosis and hepatocellular carcinoma. Increased hepatic iron stores are also observed in about one-third of adult NAFLD patients, though to a lesser extent than in hemochromatosis [11]. In NAFLD, iron potentiates the onset and progression of the disease by increasing ROS and altering insulin signaling and lipid metabolism, and is thought to be involved in the transition of NAFLD to NASH [24].

Unfortunately, iron deposition in the liver could not be detected in the MCD-induced NASH model used in our previous study [15]. Kirsch et al. reported a rat model of MCD with iron-overload [25]. In that model, hepatic iron-overload worsened hepatic inflammation and fibrosis. In the present study, we applied their protocol to mice, in part because it may be useful in a future analysis of genetically engineered mice. Histological examination of the liver in this model confirmed that we successfully generated an iron deposition model; iron deposition was already detectable around the portal vein after 1 week on the MCD-Fe diet. Iron deposition was not detectable in mice on a normal diet, which suggests that MCD-Fe-induced hepatocyte damage may disrupt excretion of excess iron from the liver. Although, iron deposition was limited to the area around the portal vein during the first 3 weeks, the area of iron deposition enlarged to include other areas of the parenchyma during weeks 4–12. This suggests that proper excretion of iron from the body was preserved for the first 3 weeks, after which the disruption of iron homeostasis became more pronounced, leading to diffusely distributed iron deposition in the liver at later times. Similarly, in the NASH model caused by the MCD diet, the progression of NASH pathology due to fibrosis was confirmed after 8 weeks [26].

The urinary iron concentration reached its peak at 2 weeks and then decreased to the control level. By contrast, biliary iron excretion was continuously elevated throughout the study, suggesting iron excretion from the liver to the bile is sustained to some degree into the later stages of the disease model. Similarly, serum hepcidin levels were continuously elevated throughout the 12-week period mice were fed the MCD-Fe diet. Hepcidin is a peptide hormone and a key regulator of iron metabolism in mammals that accelerates iron excretion from the body. As the pattern of hepcidin elevation was similar to that of the biliary iron concentration, we suggest hepcidin is upregulated in response to the hepatic iron accumulation. The continuous elevation in hepcidin means adaptation to iron-overload is somewhat preserved until the later disease stages in this model. Nonetheless, increased iron deposition was observed in mice after 4 weeks on the MCD-Fe diet, which suggests this adaptive mechanism cannot offset prolonged accumulation of iron.

Excessively accumulated iron is phagocytosed by liver Kupffer cells and macrophages [27]. In mice on the MCD-Fe diet, F4/80-positive macrophages were detected along the sinusoids within 1 week, and the macrophage accumulation then expanded from the portal triad into peripheral regions such that the macrophage distribution paralleled that of iron deposition. This suggests that the macrophage accumulation was a compensatory response to the iron deposition, and that the macrophages were there to phagocytose excess iron. On the other hand, macrophage accumulation can lead to chronic inflammation and organ damage. The greatly enhanced macrophage accumulation seen after 8 or 12 weeks in the model may thus reflect the development of chronic inflammation. Consistent with that idea, in mice on the MCD-Fe diet, real-time PCR analysis revealed increased expression of genes related to inflammation, oxidative stress and fibrosis.

HPE has long been prescribed clinically to treat chronic hepatitis, liver cirrhosis and other hepatic diseases. Shimokobe et al. reported that HPE is effective in NASH patients who were unresponsive to lifestyle intervention [14]. We also reported the beneficial effect of HPE on the pathology of NASH in our model [15]. In the present study, HPE treatment led to a marked reduction in hepatic iron deposition in mice on the MCD-Fe diet. This reduction was associated with enhanced biliary iron excretion. Also reduced by HPE treatment were the hepatic macrophage accumulation and the serum oxidation-reduction potential, which indicates HPE treatment significantly reduced inflammation and oxidative stress. HPE treatment thus appears to effectively ameliorate iron overload-induced liver injury.

The precise mechanism underlying the beneficial effects of HPE on iron overload-induced liver injury remains to be clarified. We speculate that the beneficial effects do not reflect the action of a single molecule or pathway, but are instead associated with the combined actions of multiple bioactive molecules active within various pathways. It was recently reported that HPE exerts a protective effect against hepatocyte apoptosis by reducing oxidative stress and maintaining cell homeostasis. The underlying mechanisms may be associated with a reduction in endoplasmic reticulum stress [28]. HPE also exerts direct inhibitory effects on the pro-inflammatory mediators nitric oxide, TNF-α and cyclooxygenase-2 in lipopolysaccharide-stimulated RAW264.7 macrophages [29]. In the present study, we confirmed that HPE suppresses inflammation in the liver. We speculate that summation of the anti-inflammatory, anti-oxidative stress and anti-apoptotic effects of HPE all contribute to the recovery of liver function and proper iron metabolism. Iron reduction therapies, such as phlebotomy and an iron-restricted diet, are now used with chronic hepatitis C patients for the purpose of reducing iron overload. Iron reduction therapy is also effective in NASH patients [30]. In addition, there is persuasive evidence that iron reduction decreases insulin resistance, and it likely also decreases oxidative stress, two key pathogenic features of NASH. By improving iron metabolism, HPE treatment may be an effective strategy that can be used as an alternative or an addition to iron reduction therapy in the treatment of NASH.

## Declarations

### Author contribution statement

Akihiro Yamauchi: Conceived and designed the experiments; Performed the experiments; Analyzed and interpreted the data; Contributed reagents, materials, analysis tools or data; Wrote the paper.

Akiko Kamiyoshi, Takayuki Sakurai: Performed the experiments.

Hiroyuki Miyazaki, Eiichi Hirano, Hong-Seok Lim, Taiichi Kaku: Performed the experiments; Contributed reagents, materials, analysis tools or data.

Takayuki Shindo: Conceived and designed the experiments; Analyzed and interpreted the data.

### Funding statement

This work was supported by Japan Bio Products Co., Ltd., as a collaborative project.

### Competing interest statement

The authors declare the following conflict of interests: Taiichi Kaku is a stockholder of Japan Bio Products Co., Ltd.Akihiro Yamauchi, Hiroyuki Miyazaki, Eiichi Hirano and Hong Seok Lim are employees of Japan Bio Products Co., Ltd.

### Additional information

No additional information is available for this paper.
